# *In vitro* toxicity evaluation of graphene oxide and reduced graphene oxide on Caco-2 cells

**DOI:** 10.1016/j.toxrep.2022.05.010

**Published:** 2022-05-17

**Authors:** O. Cebadero-Domínguez, B. Ferrández-Gómez, S. Sánchez-Ballester, J. Moreno, A. Jos, AM Cameán

**Affiliations:** a Area of Toxicology. Faculty of Pharmacy. Universidad de Sevilla, Profesor García González Nº2, Sevilla, Spain; b Packaging, Transport and Logistic Research Institute, Albert Einstein 1, Paterna, 46980 Valencia, Spain; c Area of Cellular Biology, Faculty of Biology, Universidad de Sevilla, Avda, Reina Mercedes s/n, Sevilla, Spain

**Keywords:** Graphene oxide, Reduced graphene oxide, Toxicity, Caco-2 cells, in vitro

## Abstract

Graphene derivatives are expected to have a great impact in a wide range of applications, among them as food packaging materials. This is one of the sources of potential human oral exposure to them. However, studies devoted to investigating their putative toxic effects at the intestinal level are underrepresented in the scientific literature. Thus, this study aimed to investigate the in vitro toxicity of reduced graphene oxide (rGO) and graphene oxide (GO) in the human intestinal Caco-2 cell line. rGO and GO were firstly characterized and later, cell viability was assessed after exposure to 0–250 µg/mL rGO/GO for 24 and 48 h. Internalization was evidenced for both materials using transmission electron microscopy. A mean effective concentration (24 h) of 176.3 ± 7.6 µg/mL for cytotoxicity was obtained for rGO, whereas GO did not induce any change at the concentration range evaluated. However, both of them altered oxidative stress biomarkers, causing increased reactive oxygen species (ROS) and depletion of the glutathione content (GSH) after exposures up to 24 h. Further studies, particularly with rGO, are required to elucidate their toxicity profile in experimental models relevant for oral exposures.

## Highlights

• GO and rGO are internalized by Caco-2 cells.
• rGO was cytotoxic and GO did not affect cell viability at the concentrations assayed.
• rGO and GO induced oxidative stress.

## Graphical Abstract

**Figure:**
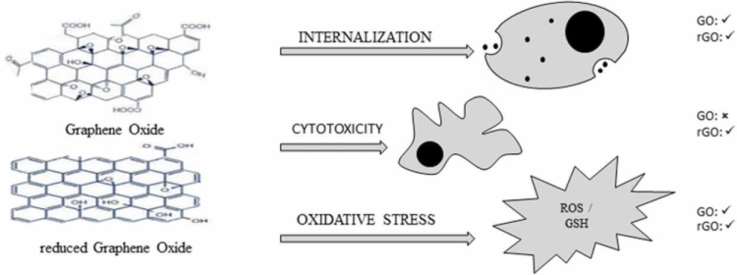

## 1 Introduction

Graphene is a monolayer of carbon atoms packed into a two dimensional (2D) honeycomb lattice [1]. The unique physical and chemical properties that both, graphene and their derivatives, possess have attracted attention in different fields including biomedicine [2], [3], pharmaceutical applications [4], biotechnology [5] or food packaging [6], [7]. Regarding food packaging applications, the use of biodegradable polymers could be an excellent alternative to petroleum-based plastics employed in the food packaging industry [8]. Polysaccharides, lipids, and proteins are some biopolymers usually employed as packaging materials [9]. However, these materials are limited due to their poor thermal stability and mechanical barrier properties, which are needed to maintain food safety and quality [7]. The interaction between graphene derivatives and some biopolymers in order to enhance their properties has been investigated [6]. Thus, it has been shown that biopolymer-based composites with graphene derivatives enhanced impermeability to gases, water and ultraviolet light resistance, reduced water solubility, and increased hydrophilicity without losing their biodegradable properties [7].

Graphene oxide (GO) is commonly used for graphene-based applications in a wide range of different fields [10]. The most popular methods used for graphene oxide preparations are based on a chemical reaction between graphite powder and strong oxidants and acids. The result of this oxidation is a graphene sheet bonded with oxygen groups in the form of carbonyl (C=O), carboxylic acid (–COOH), hydroxyl (–OH), alkoxy (C–O–C), and other oxygen-based functional groups [11]. Compared with other graphene derivatives, GO can provide a better aqueous dispersibility and colloidal stability due to the oxygenated group, suggesting advantages for biological applications [12]. In addition, GO can be reduced by a thermal or chemical procedure to synthesize reduced graphene oxide (rGO). The aim of removing the oxygen functional groups is to obtain properties closer to pristine graphene [13]. Particularly for food packaging applications, rGO-composite films have been shown to have better barrier properties than GO-films, with great potential in realizing a versatile and effective food packaging [6].

Considering that the use of nanomaterials has increased in the last years, their potential impacts on human health have also increased as a consequence of higher exposure. These nanomaterials could provide many benefits to food packaging industries, but before the application of these materials become a reality, it is required to know their human risks, and therefore their toxic effects. Thus, the European Food Safety Authority (EFSA) has published a “Guidance on risk assessment of nanomaterials to be applied in the food and feed chain: human and animal health’’ [14] that includes a tiered approach for the evaluation of nanomaterials. According to this guidance, the first step should be a physico-chemical characterization, and among the toxicity testing required, in vitro studies remain at the basal level, as they can provide insights into a nanomaterial’s hazard and its mode of action. Physico-chemical properties, such as surface charge, size, composition or structure have been demonstrated to have effects on the uptake of graphene materials by cells [15]. Moreover, they can affect the outcome of the risk assessment, as toxicity may change. Regarding in vitro toxicity testing, most of the studies have been performed in pulmonary cells [16] as inhalation is the main exposure route to graphene materials [17]. But they have been shown to induce toxic effects in a wide diversity of cell types [18], [19], [20]. However, the oral pathway is of importance when potential food and feed applications are considered, and nonetheless, the toxic effects on the gastrointestinal tract have been scarcely investigated.

Regarding toxicity at intestinal level, the Caco-2 cell line, derived from a human colon adenocarcinoma, is commonly used as an in vitro model to evaluate the toxicity of xenobiotics after oral exposure [21]. Among the scarce reports available in this model system, GO has shown to induce no or mild cytotoxicity [22], [23] meanwhile, rGO was cytotoxic in the only study available [23] with dependence on the exposure time. Oxidative stress has been suggested as a toxicity mechanism for nanomaterials in general [14] including graphene derivatives [24], [25] but for GO and rGO data are very limited in this experimental model. Thus, GO and rGO have shown to induce reactive oxygen species (ROS) in different cell types [26], [27] whereas this effect on intestinal cells was only explored by Kucki et al. [28], Domenech et al. [29], and Garriga et al. [23] with different outcomes for GO. rGO effects and other oxidative stress biomarkers were even less explored in the scientific literature.

Thus, considering that there are potential applications that require a thorough knowledge of the oral toxicity profile of graphene derivatives, the research on this topic if worth of research, particularly for rGO, with a more limited background information.

Hence, the aim of this work was to identify the hazards of two graphene derivatives, a commercial rGO and GO developed by the Technological Institute of Packaging, Transport and Logistics (ITENE). For this purpose, both graphene materials were characterized, and internalization, cytotoxicity and oxidative stress were assessed on intestinal cells of human origin (Caco-2).

## 2 Materials and methods

### 2.1 Chemicals and reagents

Cell culture reagents were provided by Gibco (Biomol, Sevilla, Spain). rGO was purchased from Graphitene, Ltd (Flixborough, UK). For the synthesis of GO, sodium nitrate (NaNO_3_) and potassium permanganate (KMnO_4_) were purchased in Sigma-Aldrich (Madrid, Spain), and hydrogen peroxide (H_2_O_2_, 30% w/w), sulfuric acid (H_2_SO_4_, 95%), ethanol absolute (EtOH) and hydrochloric acid (HCl, 37%) in Scharlab (Barcelona, Spain). Chemicals for the different assays were obtained from Sigma-Aldrich (Madrid, Spain) and VWR International Eurolab (Barcelona, Spain).

### 2.2 Synthesis and characterization

GO was synthesized by ITENE from the oxidation of graphite powder using the Modified Hummers Method (MHM) [30] as described by Sánchez Ballester [31].

Both graphene materials were characterized by X-ray diffraction spectroscopy (XRD), X-ray photoelectron spectroscopy (XPS), ζ potential, Fourier transform infrared spectroscopy (FTIR), Scanning electron microscopy (SEM) and Transmission electron microscopy (TEM). A Leybold-Hereus mod. LHS-10/20 analyzer was used to perform the XPS analysis with monochromatized Al Kα radiation (1486.6 eV) at the XPS Service of the Centro de Investigación, Tecnología e Innovación (CITIUS) of the University of Sevilla. CasaXPS software was used for the deconvolution of individual spectral peaks. The XRD spectra were obtained by an X-ray diffractometer Bruker D8 Advance A25 (Bruker, Germany) from the RX Service (CITIUS). A semi-quantitative analysis was used in the 2θ range of 3–70°. Continuous scan mode was used at 0.015°/ 0.1 s scan speed. ζ potential was measured by Malvern, Zetasizer Nano ZS available in the Functional Characterization Service (CITIUS). The samples were dispersed in milli-Q water and cell culture medium (CCM) at a concentration of 100 µg/mL. Samples were sonicated for 1 h to reduce particle agglomeration. The test was performed in triplicate. To determine their morphology, GO and rGO were dispersed in milli-Q water and cell culture medium at 100 µg/mL and sonicated for 1 h. SEM images were obtained by Zeiss EVO microscope and TEM images by a Zeiss Libra 120 microscope available at the Microscopy Service (CITIUS). *In situ* Fourier-transform infrared (FTIR) spectroscopy experiments were carried out in a Bruker Tensor 27 IR spectrometer (Bruker, Germany), available in ITENE facilities, in the range of 4000–600 cm^−1^ using the attenuated total reflectance (ATR) mode at a resolution of 4 cm^−1^ and 64 scans were performed. Backgrounds spectra were collected before each series of experiments in order to eliminate any interference from the environment.

### 2.3 Model system

The Caco-2 cell line derived from a human colon carcinoma (ATCC® HTB-37™) was maintained at 37 ºC in an atmosphere containing 5% CO_2_ at 95% relative humidity (CO_2_ incubator, Nuaire®, Spain) at the Biology Service (CITIUS). Caco-2 cells were cultured as described by Houtman et al. [32] and experiments were performed with cultures at passages 5–20. This cell line was selected as it is a well characterized model of the intestinal epithelium [33] commonly used in toxicity studies. Moreover, Tarantini et al. [34] suggested the use of undifferentiated cells because of their higher sensitivity towards nanoparticles compared to differentiated ones.

### 2.4 Internalization and cytotoxicity

To check the graphene derivatives uptake by Caco-2 cells, the cells were exposed to a suspension of GO and rGO at 100 µg/mL concentration for 24 and 48 h.

Graphene samples were sonicated for 1 h previously to the exposure. Later, cells were washed with phosphate-buffered saline (PBS) three times and fixed with 2.5% glutaraldehyde, 2% paraformaldehyde in 0.1 M cacodylate buffer (pH 7.2) for 2 h. Samples were washed thrice with 0.1 M cacodylate buffer and dehydrated in a graded ethanol series. Samples were embedded in epoxy resin and cut by ultramicrotome. Images were analyzed in a Zeiss Libra 120 TEM microscope at the Microscopy Service (CITIUS).

For the cytotoxicity tests, Caco-2 cells were seeded in 96-well culture plates at a density of 7.5 × 10^5^ cells/well. After 24 h, cells were incubated with 200 μL of rGO and GO solutions at different concentrations (0, 1.95, 3.9, 7.81, 15.6, 31.2, 62.5, 125, and 250 µg/mL) for 24 and 48 h. Previously, the solutions were dispersed in cell culture medium by means of 1 h ultrasonic treatment (Hielscher Ultrasound Technology, Telow, Germany). MTS (3-(4,5-dimethylthiazol-2-yl)− 5-(3-carboxymethoxyphenyl)-2-(4-sulfophenyl)− 2 H-tetrazolium salt) reduction and protein content (PC) were measured as basal cytotoxicity endpoints, following the protocols described by [35] and [36], respectively.

Moreover, to avoid potential dye interferences, the method described by Liao et al. [37] was followed.

### 2.5 Oxidative stress assays

The concentrations for the oxidative stress assays were selected according to the cytotoxicity tests results. Thus, for rGO, the mean effective concentration (EC_50_) at 24 h obtained in the MTS assay (176.3 ± 7.6 µg/mL) was chosen as the higher exposure concentration, along with the fractions EC_50/2_ and EC_50/4_. GO did not show cytotoxicity, consequently, 250 µg/mL was used as the highest concentration. Cells were exposed for 4, 8, 12 and 24 h.

ROS content was measured following the method described in [38]·H_2_O_2_ at 100 µM was used as positive control and culture medium as a negative control.

Levels of GSH were determined according to [39]. Buthionine sulfoximine (BSO) 300 µM was used as positive control and culture medium as negative control.

### 2.6 Calculations and statistical analysis

Data for cytotoxicity and oxidative stress parameters were expressed as mean ± standard deviation (SD) in relation to control. The Kolmogorov-Smirnov test was used to confirm the normality of the distribution and the homogeneity of variances. Statistical analysis was carried out using the Kruskal Wallis test followed by Dunn’s multiple comparison test for data that did not follow a normal distribution and one-way ANOVA, followed by Tukey's multiple comparisons test for data with a normal distribution. All analysis were performed with Graph-Pad Prisma 9 version 9.0.0 software. Differences were considered significant at *p < 0.05, * *p < 0.01 and * **p < 0.001. EC_50_ value was derived by linear regression in the concentration-response curve. All experiments were performed at least three times.

## 3 Results

### 3.1 Characterization

**Fig. 1:**
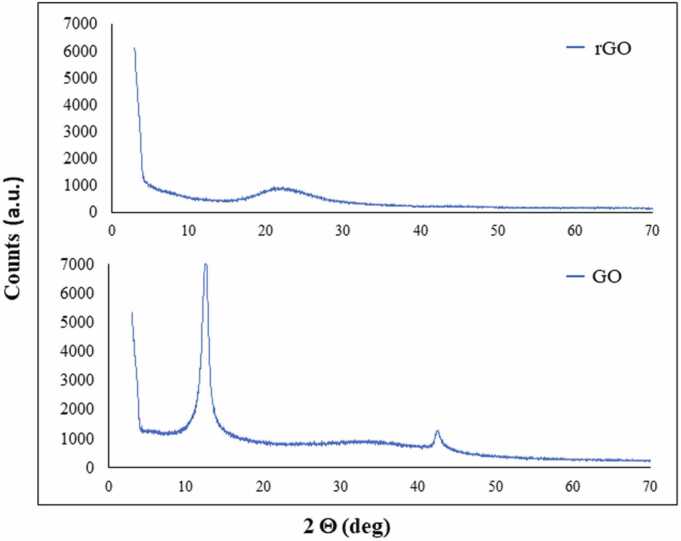
X-ray diffraction spectroscopy (XRD) of rGO and GO.

Fig. 1 shows the XRD spectra of rGO and GO. Literature results report the characteristic diffraction peak of graphite at 2θ = 26.7º, with a distance between layers of 0.34 nm, indicating a highly organized crystal structure [40]. After the chemical oxidation of graphite, the value of 2-theta of the main peak is shifted to 12.6º, which indicates that the interlayer spacing was extended due to the intercalation of the oxygen-containing functional groups into the layers, increasing the interlayer distance to 0.75 nm. A second diffraction peak at higher values of 2-theta (2θ = 42.5º) can be observed in the XRD spectra of GO, denoting a short-range order in stacked graphene layers [41]. In contrast, the XRD pattern of rGO shows a broad peak at approximately 2θ = 21.5º with an interlayer distance of 0.32 nm, indicating a slight difference when compared to the graphite (0.34 nm). These findings demonstrated that the crystalline structure can be restored after the reduction process of GO.

**Fig. 2:**
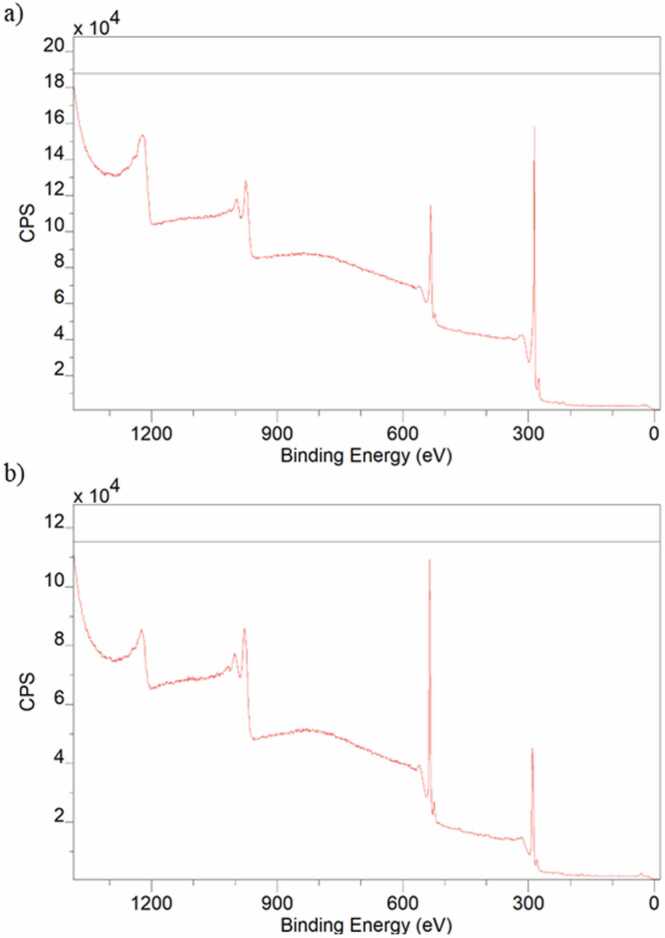
X-ray photoelectron spectra of rGO (a) and GO (b).

The elements and composition constituting the graphene samples were determined by XPS. As shown in Fig. 2, the atomic content of rGO (Fig. 2a) revealed oxygen content (13.6 at%) and carbon content (86.3 at%). As expected, oxygen content increased (33.16 at%) and carbon content decreased (66.26 at%) in the GO sample (Fig. 2b).

ζ potential was measured to determine the surface charge and aggregation state of the tested solutions. A ζ potential between ± 30 mV is considered as moderate stability. However, values close to zero may mean particle aggregation [42]. The ζ potentials of the samples in milli-Q water and cell culture medium are shown in Table 1. ζ potentials were negative for both. GO demonstrate higher stability in milli Q water. Nevertheless, both graphene derivatives showed values nearer to zero in cell culture medium, suggesting a lower stability behavior in this case.

**Table 1:** ζ-potential measurement of rGO and GO at 100 µg/mL.

	***ζ-*****potential (mV)**	
	Milli-Q water	Cell Culture Medium
**rGO**	-17.4 ± 0.4	-15.8 ± 2.5
**GO**	-30.3 ± 0.6	-10.9 ± 0.3

**Fig. 3:**
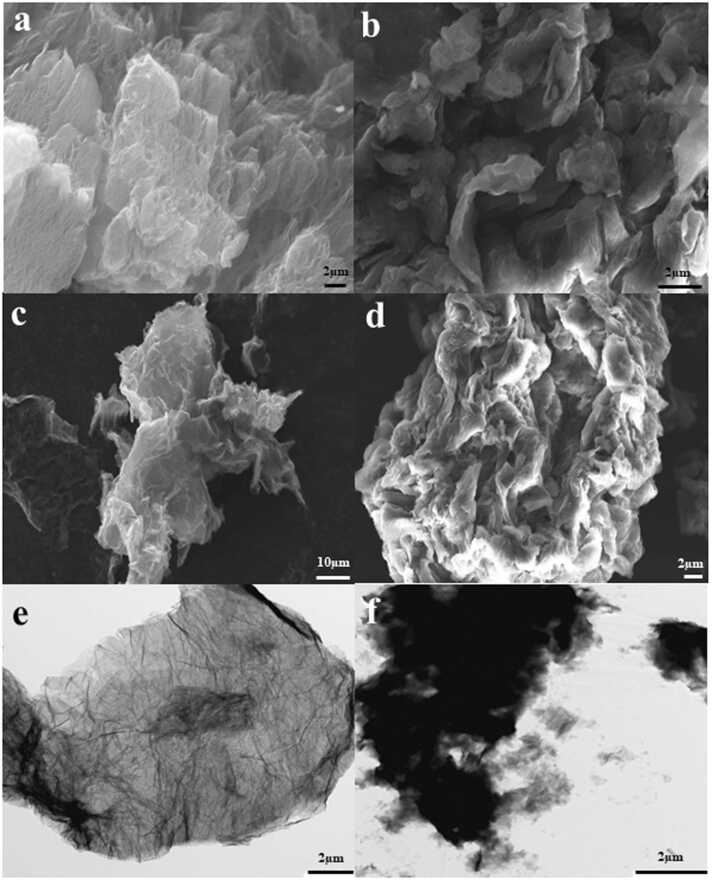
SEM images (a,b,c,d) of rGO powder (a), rGO aqueous dispersion (c), GO powder (b) and GO aqueous dispersion (d). TEM images of rGO (e) and GO (f).

The morphological structure of rGO and GO was determined by SEM and TEM (Fig. 3). The rGO (Fig. 3a) and GO (Fig. 3b) powder images observed by SEM had a few irregular layers and wrinkled structures. Both graphene-based materials created agglomerates in aqueous dispersion (Fig. 3c, d). The TEM images revealed higher transparency areas of rGO due to much thinner layers than GO. Dark areas of GO images indicate layer stacking (e, f).

**Fig. 4:**
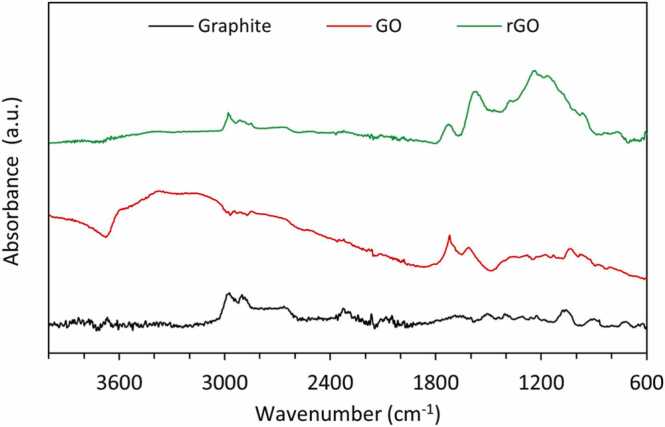
FTIR spectra of rGO and GO.

Fig. 4 shows the FTIR spectra of graphite, GO, and rGO. While no significant peaks attributable to oxygen-containing groups were observed in raw graphite [43]. GO was found to exhibit the characteristic absorption peaks of oxygen-containing groups formed during the oxidation of graphite to graphene oxide.

The GO absorption peaks appearing at 1720, 1650 and 1050 cm^−1^ can be assigned to the carboxyl stretching vibration (-O-C=O), the carbonyl stretching vibration (C=O), and the epoxide group (C-O-C), respectively, while the peak attributed to the unoxidized graphitic domains is presented at 1625 cm^−1^ [44]. Additionally, the band present between 3100 and 3400 cm^−1^ can be assigned to the hydroxyl stretching vibration (O-H). These results are in agreement with literature data [45], confirming that the oxidation of the graphite to GO was conducted successfully.

Regarding the rGO sample, it is important to highlight that the characteristic peaks could be identified at 1720 (C=O stretching), 1580 (C=C stretching), and 1190 cm-1 (C−OH stretching). However, the stretching vibration of C-OH and C=O bonds in rGO was decreased or disappeared compared to GO, which are caused by the fact that oxygen-containing groups attached to the GO are removed during the reduction process [46], [47].

### 3.2 Internalization and cytotoxicity assays

**Fig. 5:**
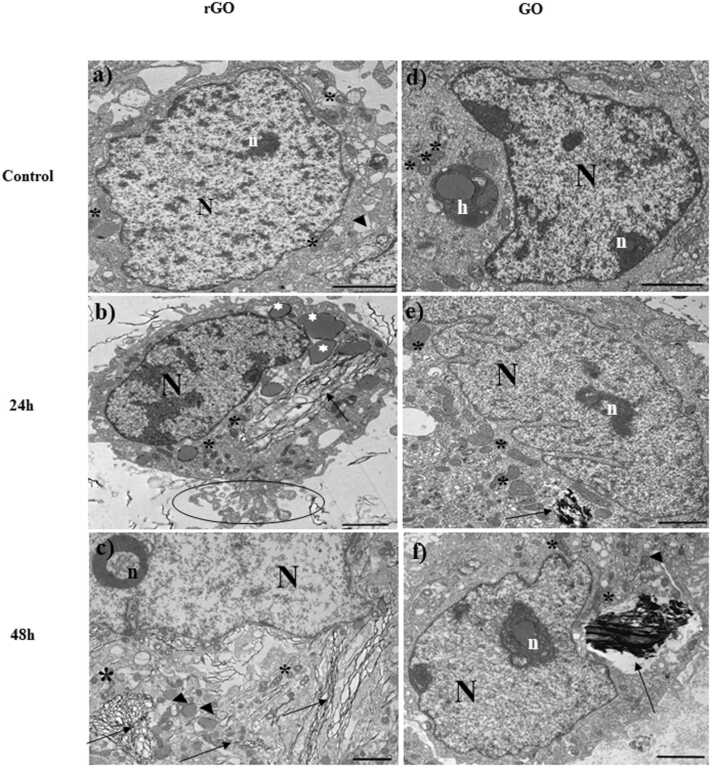
TEM images of cellular internalization of rGO and GO in Caco-2 cells. Unexposed control cells (a,d), Caco-2 exposed to rGO after 24 h (b) and 48 h (c) and exposed to GO after 24 h (e) and 48 h (f) at 100 μg/mL. h heterophagosome, N nucleus, n nucleolus, * mitochondria, ▲endoplasmic reticulum, ìdense bodies, apoptotic bodies (circle) and graphene materials (black narrows). Scale bar: 2 µm.

Fig. 5 shows the interaction of Caco-2 cells with graphene materials. On non-exposed cells (Fig. 5a, d), regular nuclei with pockets and tunnels were observed. Free ribosomes, mitochondria and cisternae of the endoplasmic reticulum were found in the cytoplasm of both control samples.

Cells exposed to rGO for 24 h (Fig. 5b), showed an increase of heterochromatin in the nucleus. In the cytoplasm, dense bodies were identified, probably derived from the endoplasmic reticulum. An increased density of mitochondria was observed. Cells evidenced a fragmentation into apoptotic bodies. Images demonstrated the presence of rGO inside cells. In cells treated with GO for 24 h (Fig. 5e) more irregular and heterochromatic nucleus than in cells exposed to rGO were localized. Mitochondria are better conserved in comparison to rGO exposed cells. In the same way, GO was internalized by Caco-2 cells.

In Fig. 5c, the segregation of the nucleolus is shown. Mitochondria appear with dense matrix and dilated cristae that indicate alterations of cells. The endoplasmatic reticulum appears as a dense body. rGO was internalized by Caco-2 cells also after 48 h of exposure. GO was localized inside a vesicle (Fig. 5f) and mitochondria were more altered than after 24 h.

**Fig. 6:**
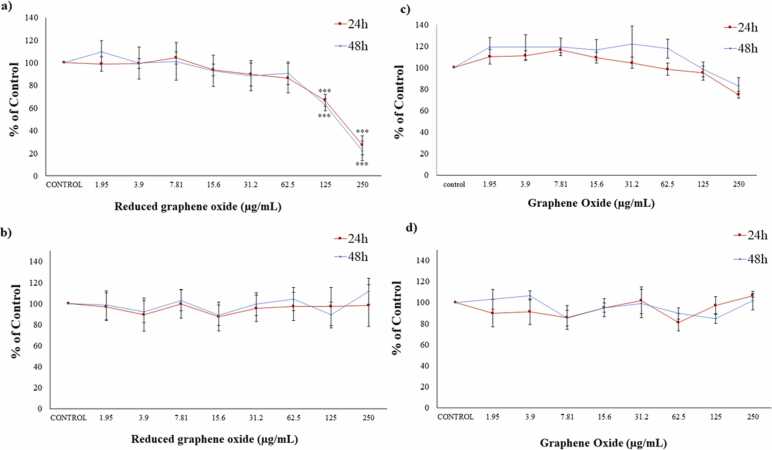
Reduction of tetrazolium salt (a, c) and protein content (b, d) of Caco-2 cells after 24 h and 48 h of exposure to 0–250 μg/mL rGO (a, b) and GO (c, d). All values are expressed as mean ± SD. * ** Significantly different from control (p < 0.001).

In relation to cytotoxicity assays, Caco-2 cultures treated with rGO showed significant changes with respect to the control cells from 125 µg/mL after 24 h and 48 h of exposure in MTS reduction (Fig. 6a). Nonetheless, PC did not show significant changes at any of the conditions assayed (Fig. 6b). Thus, the EC_50_ (MTS) values obtained were 176.3 ± 7.6 µg/mL for 24 h and 166.5 ± 21.9 µg/mL for 48 h exposures.

Moreover, Caco-2 cells exposed to GO did not undergo a significant reduction in the endpoints considered at the conditions tested (Fig. 6c, d).

### 3.3 Oxidative stress

**Fig. 7:**
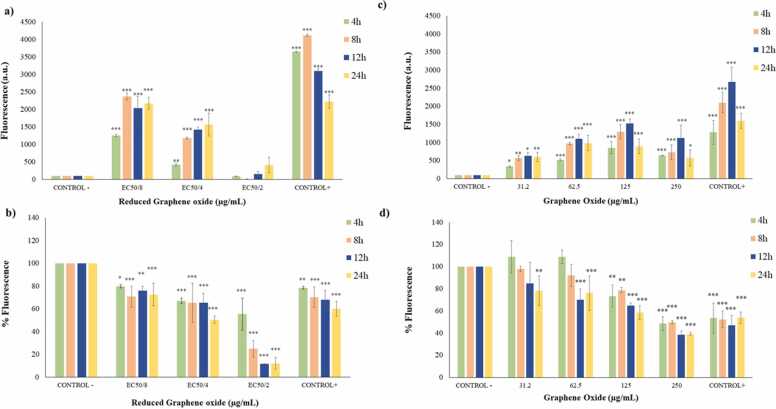
Reactive oxygen species (ROS) levels on Caco-2 cells after 4, 8, 12 and 24 h of exposure to EC_50/8_, EC_50/4,_ and EC_50/2_ rGO (a) and to 31.2, 62.5, 125 and 250 µg/mL GO (c). Reduced glutathione (GSH) levels on Caco-2 cells after 4, 8, 12 and 24 h of exposure to EC_50/8_, EC_50/4_ and EC_50/2_ rGO (b) and to 31.2, 62.5, 125 and 250 µg/mL GO (d). All values are expressed as mean ± s.d. * P < 0.05, * * P < 0.01 and * **P < 0.001 significantly different from control group.

According to Fig. 7, rGO increased ROS levels after any exposure time (Fig. 7a). The highest values were observed at EC_50/8_ concentration. Moreover, GSH content was significantly depleted when Caco-2 cultures were exposed to rGO in a time and concentration dependent way (Fig. 7b).

Caco-2 cells underwent a ROS level increment at GO testing conditions (Fig. 7c). Regarding GSH content, at the lower concentrations, no significant changes were observed after exposure for 4 and 8 h. However, GSH decreased at these concentrations after 12 and 24 h of exposure. These results demonstrated that Caco-2 cultures experimented a GSH level reduction in a time and concentration dependent way (Fig. 7d).

## 4 Discussion

The safety of graphene materials is a controversial issue that has been the focus of research due to the wide range of their potential applications and hazards [48]. However, the research on their effects on the gastrointestinal system is hugely underrepresented in the current scientific literature in comparison to other exposure routes. Thus, research on the intestinal toxicity of graphene materials is of interest. There are two main sources of potential oral exposure to nanomaterials: (a) direct ingestion of materials present in food or released from food packaging and (b) indirect ingestion of inhaled materials [49]. Moreover, uptake of graphene materials by cells have been previously reported [28], [50], [51]. Endocytosis is the mechanism suggested by the scientific literature for graphene related materials [15], [51], [52] and it is influenced by particle size and surface chemistry [51]. Our results have shown that both, rGO and GO are internalized by Caco-2 cells. Other cell lines such as HepG2 (human hepatocarcinoma) has been reported to uptake GO but not rGO due to its higher hydrophobicity [24]. On the contrary, GO and rGO were taken up effectively by A549 cells (from human lung) [53], [54], [55] observed that the internalization of GO was highly dependent on the cell differentiation status of the intestinal cells. Thus, even large GO sheets were uptaken by undifferentiated Caco-2 cells, whereas no uptake could be found for differentiated cells.However, oral in vivo studies with GO in rats have reported kidney damage [56], indicative of systemic effects and therefore of uptake. Also, in a biodistribution study of GO labelled with ^125^I in mice orally exposed, Zhang et al. [57] found radioactivity in plenty of organs, mainly the liver. A revision of toxicity studies with graphene materials including in vivo oral experiments has been recently published by our research group [58]. Thus, their intestinal absorption is confirmed. The main cell morphological features altered by rGO in Caco-2 cells were mitochondria and the appearance of apoptotic bodies, in agreement with cytotoxicity results obtained. The absence of morphological damage after exposure to GO was also observed by Kucki et al. [28] with no data reported for rGO.

Regarding cytotoxicity, very different results have been obtained for both graphene derivatives. Thus, in the same concentration range (0–250 µg/mL), rGO reduced cell viability in a significant way as measured by the MTS assay, whereas GO did not show any variation. This is in agreement with the mild or no cytotoxicity reported in the scientific literature for GO in Caco-2 cells [22], [23], [28], [29], at concentrations up to 500 µg/mL. On the contrary, the only study that also evaluated the cytotoxicity of rGO in Caco-2 cultures showed toxic effects [23] in agreement with our results. These authors found no effects after 24 h exposure to 3 µg/mL rGO, but a significant cell viability reduction after 72 h using the MTT test. In the present study, decreased viability was only evident with the MTS assay and this could give a hint of the toxicity mechanisms involved. Thus, in this test the tetrazolium salt is bioreduced by mitochondrial dehydrogenase enzymes in metabolically active cells, making this endpoint a good biomarker for the disturbances induced in this organelle [32]. The cell viability reduction observed is in agreement with the mitochondria affectation observed by TEM.

Oxidative stress parameters (ROS and GSH) have been altered with independence of the cytotoxicity tests results. ROS are highly reactive substances that play several physiological roles (such as cell signaling). They are normally generated as by-products of oxygen metabolism, but xenobiotics among other factors can contribute to greatly increase their production [59]. Kucki et al. [28] found an increase in ROS levels induced by GO in Caco-2 cultures (up to 40 µg/mL, 2 h exposure), in agreement with our results. On the contrary, Domenech et al. [29] and Garriga et al. [23] reported no changes. These last authors were the only ones that also evaluated rGO, for which they found increased ROS values (3 µg/mL, 24 h exposure). The observed increase in ROS values could be attributed to the penetration of sharp-edged graphene particles into biological membranes, impairing intracellular organelles and increasing the production of free radicals by the harmed cells as suggested by different authors [26], [27]. Surprisingly, another common oxidative stress parameter such as GSH levels have not been investigated in Caco-2 cultures exposed to graphene derivatives. Both, rGO and GO reduced GSH content. GSH is the main low-molecular-weight thiol-containing peptide present in most living cells and helps to prevent oxidative stress through the removal of ROS. Thus, to some extent, ROS formation can be outbalanced by the cellular antioxidant defense. Oxidative stress occurs when this critical balance is disrupted due to depletion of antioxidant or excess accumulation of ROS, or both. In this case, both, ROS increase and GSH decrease were observed for both graphene derivatives, but only rGO induced clear effects on cell viability.

Toxicity differences between rGO and GO, with GO showing lower toxicity, has been evidenced in Caco-2 cultures only by Garriga et al. [23], but also in other cell types such as the MCF-7 cell line (human breast adenocarcinoma), or glioblastoma cells [60]. This has been explained, among other factors, due to the oxygenated functional groups on their surface that shield the hydrophobic domains [23]. Jaworski et al. [60] concluded that rGO induced cell death mostly through the apoptosis pathway. In the present study, apoptotic bodies were also observed in Caco-2 cultures treated with rGO.

This work confirms the different toxicity profiles of rGO and GO in an experimental model (Caco-2 cells) scarcely used in graphene-related research. Moreover, the limited data available of rGO at intestinal level, and their potential preferred use in food packaging applications, target further research needs for this material.

## CRediT authorship contribution statement

**O. Cebadero-Domínguez**: Investigation, Writing − original draft, Visualization. **B. Ferrández-Gómez**: Investigation, Writing − original draft, Visualization. **S. Sánchez-Ballester**: Investigation, Resources, Writing − original draft, Visualization, Writing − review & editing. **J. Moreno**: Investigation. **A. Jos**: Conceptualization, Resources, Writing − original draft, Writing − review & editing, Supervision, Project administration, Funding adquisition. **A.M. Cameán**: Resources, Writing − review & editing, Supervision, Funding adquisition.

## Declaration of Competing Interest

The authors declare that they have no known competing financial interests or personal relationships that could have appeared to influence the work reported in this paper.
